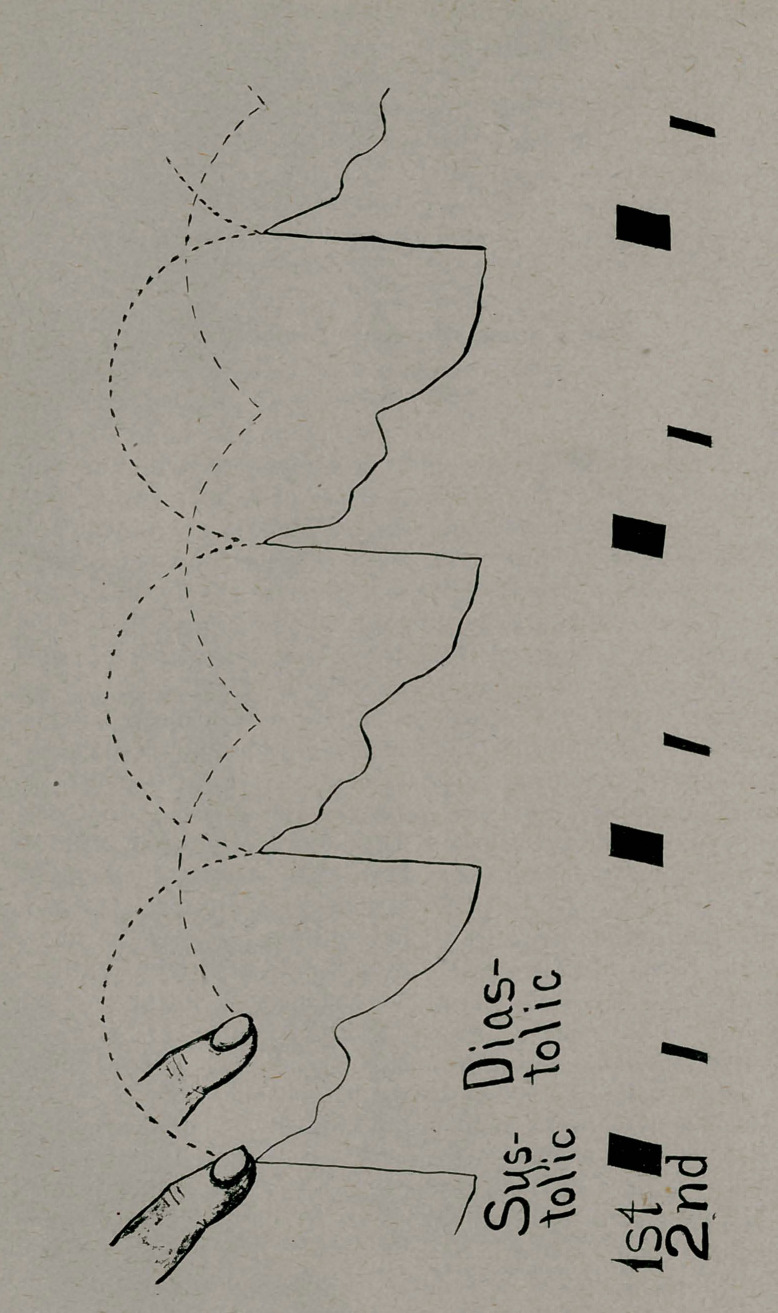# The Timing of Heart Murmurs

**Published:** 1912-02

**Authors:** Eli H. Long

**Affiliations:** Buffalo, N. Y.


### The Timing of Heart Murmurs.

ELI H. LONG, M. D.
Buffalo, N. Y.

IN determining whether a heart murmur is systolic or diastolic in time we rely largely upon a comparison with the time of the pulsations felt in the radial artery or in the carotid. We can sometimes make a more immediate comparison with the cardiac apex impulse, but often that is not palpable, so chief reliance must be upon comparison with the arterial pulse. Ordinary palpation of the pulse is usually sufficient, but when the rate is rapid or the murmur is unusual there is uncertainty and the personal equation is great.

For some years the writer has employed with much satisfaction a modification of this aid which gives absolute certainty as to the time of a murmur in most cases. It consists simply in making intermittent pressure upon the radial artery to correspond in time with the murmur, i.e., the finger is brought down upon the radial synchronously with each occurrence of the murmur to be timed and lifted away during the interval between. If this intermittent pressure occurs with the systoles the finger feels the pulsation of the artery each time; but if with the diastoles the finger feels no pulsation whatever. With this method, therefore, if the finger pressing with the murmur feels the radial pulsations the murmur must be systolic, while if the finger feels no pulsation the murmur must be diastolic.

Familiarity with this method can be easily acquired by timing the normal heart sounds in the same way. A trial will at once demonstrate the fact that the finger pressing intermittently with the first sound will feel the radial pulse, while the finger pressing with the second sound will feel no pulse. A glance at the diagram will make the matter clear. Great irregularity of the heart may defeat any attempt at timing murmurs accurately, but slight irregularity will leave sufficient regular cycles to apply this method.

## Figures and Tables

**Figure f1:**